# A multicenter, double-blind, randomized trial on the bleeding profile of a drospirenone-only pill 4 mg over nine cycles in comparison with desogestrel 0.075 mg

**DOI:** 10.1007/s00404-019-05340-4

**Published:** 2019-11-14

**Authors:** Santiago Palacios, Enrico Colli, Pedro-Antonio Regidor

**Affiliations:** 1 Instituto Palacios, Salud y Medicina de la Mujer, C/ Antonio Acuña, 9, 28009 Madrid, Spain; 2 Exeltis HealthCare Madrid, C/ Manuel Pombo Angulo 28, 4th Floor, 28050 Madrid, Spain; 3 Exeltis Europe, Adalperostr. 84, 85737 Ismaning, Germany

**Keywords:** Drospirenone only pill, Cycle control, Bleeding

## Abstract

**Purpose:**

A typical compliance problem in the use of traditional progestin-only pills is the irregular bleeding pattern and the strict daily intake. Desogestrel 75 mg has a 12-h missed-pill window; however, its poor cycle control limits a more common use.

**Methods:**

A drospirenone (DRSP)-only pill was developed to improve the bleeding profile.

**Setting:**

A phase III study in healthy women aged 18–45 years was performed to compare the bleeding profile and safety of a DRSP-only pill in a regime of 24 days of 4 mg of DRSP tablets followed by 4 days of placebo versus desogestrel 0.075 mg per day continuously over nine cycles.

**Population:**

A total of 858 women with 6691 drospirenone and 332 women with 2487 desogestrel treatment cycles were analyzed.

**Main outcome measures:**

The primary end point was the proportion of women with unscheduled bleeding/spotting in each cycle from cycles 2 to 9 and cumulative in cycles 2–4 and cycles 7–9.

**Results:**

In each cycle, up to cycle 7, the proportion of women with unscheduled bleeding was statistically significantly lower in the DRSP group than in the DSG group (*p* = 0.0001, Chi-square test).

**Conclusions:**

This report describes the improvement in bleeding profile of women using the new DRSP-only oral contraceptive in comparison to DSG, providing a better quality of live and adherence to the contraceptive method.

EudraCT Registration Number: 2011-002396-42.

**Electronic supplementary material:**

The online version of this article (10.1007/s00404-019-05340-4) contains supplementary material, which is available to authorized users.

## Introduction

Oral contraceptives are among the most popular forms of contraception. They can be divided into combined oral contraceptive pills (COCPs), and progestogen-only pills (POPs).

In comparison to COCPs, POPs offer several advantages: they are associated with a decreased venous thromboembolism (VTE) risk [1, 2] and cause fewer metabolic changes [3]. This makes them a suitable option for women who are intolerant to or contraindicated for estrogens (due to migraine or cardiovascular risk factors such as hypertension, hyperlipidemias, obesity, diabetes, smoking habits, etc.) [4, 5].

POPs provide contraceptive efficacy through various mechanisms. Regimens of the first and second generation displayed only incomplete ovulation inhibition. However, due to their additional effects on the cervical mucus and the endometrium, the efficacy is close to that of COCs. The efficacy is further enhanced by complete ovulation inhibition, but a poor cycle control remains a common side effect [6, 7].

The third generation of POPs introduced the inhibition of ovulation-enhancing efficacy with a pearl index like that of COC [7]. However, problematic bleeding while using POPs is challenging [7].

During a normal menstrual cycle, the endometrium is exposed to circulating sex steroids. It is the sequential exposure of the endometrium to the natural steroids, estradiol and progesterone, that leads to characteristic histological features [8].

Endometrial proliferation is a result of the effects of estradiol exposure during the follicular phase in regularly cycling women. In contrast, the physiological secretion of progesterone after ovulation results in a secretory transformation of the primary proliferated endometrium. Progesterone has not only an intrinsic effect on the endometrium, but also local and systemic antiestrogenic effects. These effects lead to an inhibition of the glandular differentiation and subsequent differentiation of the uterine endometrium. In case of no pregnancy, menstrual bleeding onset is triggered due to the estradiol and progesterone deficiency at the end of the cycle [9].

Theses mechanisms are modified when exogenous sex steroids are administered, during the use of hormonal contraceptives and/or menopausal substitution hormonal treatment. They exert not only clinical changes, but also cause endometrial histology findings [9, 10].

Hence, the mechanisms involved in bleeding-associated disorders during the use of combined hormonal contraceptives or estrogen free contraceptives are still unclear.

Some of the possible explanations of these bleeding disorders under the use of contraceptives are as follows.

The rise of the changes in the tissue perfusion in combination with local angiogenetic factors, together with a superficial blood vessel permeability and with the change of receptor functions to steroidal hormones in the endometrium, is the most accepted mode of explanation for these bleeding disorders [10].

Unscheduled bleeding or spotting still represents a contraceptive problem that will be negatively associated by women using contraceptives. This clinical feature of the problematic bleeding is therefore the most common quoted reason for discontinuation in up to 25% of users [11, 12].

A novel developed drospirenone (DRSP)-only pill has been described in a previous study [2]. The aim of the present study was to further assess the improvement in the bleeding profile of this new drospirenone-only pill containing 4 mg over nine cycles and to compare it with the standard used estrogen-free contraceptive desogestrel 0.075 mg.

## Materials and methods

This phase III study was a double-blinded, randomized controlled trial including 88 centers in Austria, Czech Republic, Germany, Hungary, Poland, Romania, Slovakia and Spain. The studies were performed between August 1, 2012 and January 27, 2014. The protocol was designed and conducted according to existing legal regulations, and in accordance with good clinical practice in the conduct of clinical trials and the Declaration of Helsinki including recommendations made in the European Medicines Agency (EMA) CHMP Guideline on Clinical Investigation of Steroid Contraceptives in Women. The studies were conducted in compliance with the principles of good clinical practice. Institutional review board approval was obtained for all study sites.

### Study medication

The study medication was DRSP, one tablet of 4 mg non-micronized DRSP per day, via oral route, with consecutive administration of 24 active tablets and 4 placebo tablets, and no tablet-free interval between two consecutive cycles.

Desogestrel 0.075 mg (in a regimen of 28 active pills, marketed under trade names such as Cerazette^®^ and Cerazet^®^) was chosen as the comparator for safety, because it is more effective in preventing ovulation than other progestogen-only pills (POP). It is also the first POP with a missed-pill window of 12 h, instead of the 3 h allowed by conventional POPs, and is one of the leading POPs on the European market.

Medication compliance was measured using an electronic diary, providing time and hour for each tablet intake, and therefore allowing for calculation of the number of intakes of study medication delayed for more than 12 h, i.e., more than 36 h after the previous tablet intake.

### Study populations

**Fig. 1:**
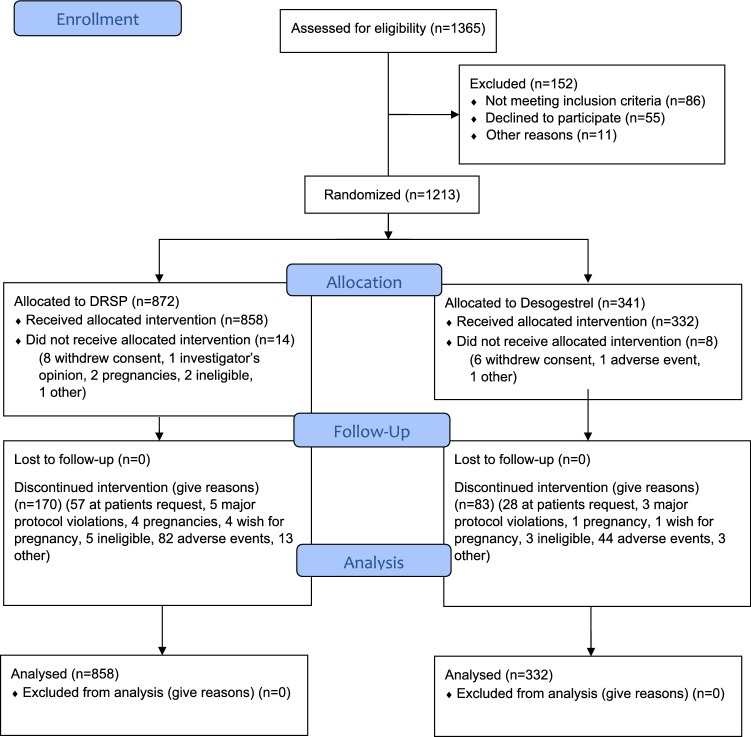
Consort of the study

A total of 858 women with 6691 drospirenone and 332 women with 2487 desogestrel treatment cycles were analyzed (see Fig. 1).

Subjects included in this study were all women of child-bearing potential, at risk of pregnancy, agreeing to use only the study medication for contraception for the duration of the study medication treatment, aged 18–45 years, with systolic blood pressure (SBP) < 140 mmHg and diastolic blood pressure (DBP) < 90 mmHg. Subjects could either start the study medication with a break of at least 1 day after the administration of another hormonal contraceptive (“starters”) or switch directly from another hormonal contraceptive to the study medication with no break in administration (“switchers”) (Table 1 depicts the clinical data).

**Table 1:** Baseline patients’ characteristics

	Statistic	Study population	
		DRSP 4 mg (*N* = 858)	Desogestrel 0.075 mg (*N* = 332)
Age (years)	Mean (SD)	28.9 (7.1)	28.9 (7.1)
Age group			
≤ 35 years	*n* (%)	682 (79.5)	259 (78.0)
> 35 years	*n* (%)	176 (20.5)	73 (22.0)
Ethnicity			
Caucasian	*n* (%)	856 (99.8)	331 (99.7)
BMI (kg/m^2^)	Mean (SD)	22.96 (3.537)	22.82 (3.905)
	Min/max	16.6/41.0	15.9/38.0
BMI group			
< 30 kg/m^2^	*n* (%)	828 (96.5)	316 (95.2)
≥ 30 kg/m^2^	*n* (%)	30 (3.5)	16 (4.8)
BP group			
SBP < 130 and DBP < 85 mmHg	*n* (%)	727 (84.7)	290 (87.3)
SBP ≥ 130 and DBP ≥ 85 mmHg	*n* (%)	131 (15.3)	42 (12.7)
Subject status			
Switcher	*n* (%)		
Direct switcher	*n* (%)	628 (73.2)	259 (78.0)
Indirect switcher	*n* (%)	39 (4.5)	14 (4.2)
Starter	*n* (%)	191 (22.3)	59 (17.8)
Unknown	*n* (%)	–	–
VTE risk factor			
Presence of at least one risk factor	*n* (%)	142 (16.5)	59 (17.8)
Previous delivery			
Yes	*n* (%)	395 (46.0)	150 (45.2)
Regular menstrual bleeding during the last 6 cycles			
Yes	*n* (%)	786 (91.6)	305 (91.9)
Prior treatment with sex hormones and modulators of the genital system			
Yes	*n* (%)	469 (54.7)	195 (58.7)

All participants gave their written informed consent for participation in the clinical trial after obtention of the correspondent ethical committee approval.

### Bleeding

Scheduled bleeding or spotting was defined as any bleeding or spotting that occurred during hormone-free intervals (defined as days 25–28 ± 1). Up to eight consecutive bleeding/spotting days were considered as scheduled bleeding days. Unscheduled bleeding or spotting day was defined as any bleeding/spotting that occurred while taking active hormones (days 2–23), except days which were classified as scheduled bleeding days. As desogestrel is administered without any free period, no scheduled bleeding is expected. Subjects recorded any vaginal bleeding or spotting by intensity (slight, moderate, heavy) per each medication cycle in an electronic diary.

### Primary efficacy end point

Proportion of women with unscheduled bleeding/spotting in each cycle from cycles 2 to 9 and cumulative in cycles 2–4 and cycles 7–9.

### Secondary efficacy end points

Number of bleeding/spotting days during cycles 2–4, 7–9 and 2–9 and proportion of subjects with no bleeding/spotting.

### Safety

Adverse events (AEs), any untoward medical occurrence in a subject, reported by the subject or observed by the clinical investigator during the study were registered using the case report form (CRF), including duration, causality assessed by investigator, seriousness, severity, frequency, treatment, action taken and outcome. Deviations from the reference ranges of laboratory parameters (thyroid function, hematology, urinalysis, biochemistry, pregnancy test) were evaluated regarding clinical significance by the investigator. Serious adverse events (SAEs) were AEs with any of the following criteria: resulted in death, were life threatening, required hospitalization, resulted in significant disability or incapacity, congenital abnormalities. Deep vein thrombosis or pulmonary embolism and hyperkalemia were considered as AEs of special interest and would lead to discontinuation. Vaginal bleeding was considered an AE if it required any additional treatment, led to discontinuation, or fulfilled a seriousness criterion (Table 2 depicts bleeding related AE’s).

**Table 2:** Early study withdrawal associated with abnormal uterine bleeding

Preferred term	DRSP 4 mg (*N* = 858) *n* (%)	Desogestrel 0.075 mg (*N* = 332) *n* (%)	Total (*N* = 1190) *n* (%)
Abnormal uterine bleeding	27 (3.2)	22 (6.6)	49 (4.2)
Dysmenorrhea	1 (0.1)	0	1 (0.1)

*N* number of subjects in the specified treatment group, *n* number of subjects with data available, % percentage based on *N*

*p* < 0.05

### Sample size

To test non-inferiority of the bleeding pattern between the two treatment groups (assuming a 24% proportion of the control group, 9% non-inferiority margin, one-sided type I error 2·5, 80% power, and 2:1 treatment allocation rate), a sample size of 531 in the DRSP group and 266 in the desogestrel group was required. To prove superiority under the same assumptions, a sample size of 443 in the DRSP group and 222 in the desogestrel group was required. Considering a possible dropout rate of 20%, to attain a 5:2 ratio, 857 DRSP and 333 desogestrel-treated women were to be enrolled.

### Statistics

The vaginal bleeding pattern statistic was performed on the FAS. Bleeding data were summarized by treatment groups by means of the default summary statistics. The hypothesis that drospirenone is non-inferior to desogestrel regarding the proportion of subjects with unscheduled bleeding/spotting during cycles 2–6 was confirmed using Chi-square test. The number and rate of subjects with different bleeding patterns was presented for each cycle and cumulatively in cycles 2–4 and cycles 7–9. Chi-square test was applied to compare the rates in both treatment groups. The numbers of bleeding/spotting days and bleeding/spotting episodes were presented by each cycle and by cycles 2–4, 7–9 and 2–9. The treatment groups were compared using a Wilcoxon rank-sum test. The numbers of missed tablets or entries in the e-diaries for subjects with unscheduled bleeding/spotting were presented by treatment cycle.

## Results

858 patients were treated with drospirenone only 4 mg during the nine cycles and 332 women were treated with desogestrel 0.075 mg.

The proportion of women with bleeding and spotting decreased from 69.7% in cycle 2 to 56.3% in cycle 9 in the DRSP-only group and from 74.0 to 45.3% in the desogestrel group; the overall median number of bleeding and spotting days decreased from 10 days (first reference period: cycles 2–4) to 6 days (last reference period: cycles 7–9) in the DRSP group and from 12 to 7 days in the DSG group. Among these, spotting days prevailed (see Table 3).

**Table 3:** Number of patients with bleeding or spotting by treatment cycle and period

Cycle	DRSP 4 mg *n*/*m* (%)	DSG 0.075 mg *n*/*m* (%)	Difference (95% CI)	Chi square test *p* value
Cycle 1	692/765 (90.5)	284/305 (93.1)	− 2.66 (− 6.18, 0.87)	0.1657
Cycle 2	482/692 (69.7)	211/285 (74.0)	− 4.38 (− 10.5; 1.75)	0.1704
Cycle 3	429/637 (67.3)	160/251 (63.7)	3.60 (− 3.37; 10.58)	0.3064
Cycle 4	390/606 (64.4)	161/244 (66.0)	− 1.63 (− 8.69; 5.44)	0.6531
Cycle 5	351/566 (62.0)	118/219 (53.9)	8.13 (0.41; 15.85)	0.0372
Cycle 6	305/530 (57.5)	110/199 (55.3)	2.27 (− 5.82; 10.36)	0.5812
Cycle 7	292/503 (58.1)	91/185 (49.2)	8.86 (0.47; 17.26)	0.0380
Cycle 8	264/468 (56.4)	87/178 (48.9)	7.53 (− 1.07; 16.14)	0.0859
Cycle 9	249/442 (56.3)	73/161 (45.3)	10.99 (2.02; 19.97)	0.0167
Cycles 2–4	421/527 (79.9)	192/222 (86.5)	− 6.60 (− 12.3; − 0.95)	0.0324
Cycles 5–7	313/423 (74.0)	106/157 (67.5)	6.48 (− 1.95; 14.91)	0.1216
Cycles 7–9	274/374 (73.3)	93/137 (67.9)	5.38 (− 3.64; 14.39)	0.2312
Cycles 2–6	346/422 (82.0)	152/172 (88.4)	− 6.38 (− 12.4; − 0.35)	0.0553
Cycles 2–9	256/305 (83.9)	102/116 (87.9)	− 4.00 (− 11.2; 3.22)	0.3044

*n* number of subjects with data available, *m* number of subjects in respective cycle, % percentage based on *m*, *CI* confidence interval

The proportion of women with unscheduled bleeding/spotting during cycles 2–6 was lower in the DRSP group than in the DSG group (73.0% vs. 88.4%), with the difference (95% CI) of − 15.4% (− 21.78%; − 8.99%) between the groups. The highest proportion of women with unscheduled bleeding or spotting was observed in cycle 2: 51.4% of the DRSP and 74.0% of the DSG group women. The incidence of unscheduled bleeding decreased over time in both groups, to 43.9% in the DRSP and 45.3% in the DSG group women in cycle 9. In each cycle, up to cycle 7, the proportion of women with unscheduled bleeding was statistically significantly lower in the DRSP group than in the DSG group (*p* = 0.0001, Chi-square test) (Table 4).

**Table 4:** Number of women with unscheduled bleeding or spotting by treatment cycle and period (FAS)

Cycle	DRSP 4 mg *n*/*m* (%)	DSG 0.075 mg *n*/*m* (%)	Difference (%) (95% CI)	Chi square test *p* value
Cycle 1	375/765 (49.0)	177/305 (58.0)	− 9.01 (− 15.59; − 2.44)	0.0077
Cycle 2	356/692 (51.4)	211/285 (74.0)	− 22.59 (− 28.90; − 16.28)	< 0.0001
Cycle 3	319/637 (50.1)	160/251 (63.7)	− 13.67 (− 20.77; − 6.56)	0.0002
Cycle 4	291/606 (48.0)	161/244 (66.0)	− 17.96 (− 25.12; − 10.81)	< 0.0001
Cycle 5	252/566 (44.5)	118/219 (53.9)	− 9.36 (− 17.13; − 1.59)	0.0185
Cycle 6	240/530 (45.3)	110/199 (55.3)	− 9.99 (− 18.10; − 1.89)	0.0161
Cycle 7	221/503 (43.9)	91/185 (49.2)	− 5.25 (− 13.66; 3.16)	0.2198
Cycle 8	202/468 (43.2)	87/178 (48.9)	− 5.71 (− 14.32; 2.89)	0.1919
Cycle 9	194/442 (43.9)	73/161 (45.3)	− 1.45 (− 10.42; 7.52)	0.7511
Cycles 2–4	358/527 (67.9)	192/222 (86.5)	− 18.55 (− 24.56; − 12.55)	< 0.0001
Cycles 5–7	269/423 (63.6)	106/157 (67.5)	− 3.92 (− 12.56; 4.72)	0.3799
Cycles 7–9	243/374 (65.0)	93/137 (67.9)	− 2.91 (− 12.10; 6.28)	0.5392
Cycles 2–6	308/422 (73.0)	152/172 (88.4)	− 15.39 (− 21.78; − 8.99)	< 0.0001
Cycles 2–9	243/305 (79.7)	102/116 (87.9)	− 8.26 (− 15.71; − 0.81)	0.0490

*n* number of subjects with data available, % percentage based on *m*, *m* number of subjects in respective cycle, *CI* confidence interval

The mean [SD] number of unscheduled bleeding and spotting days during cycles 2–9 was statistically significantly lower in the DRSP group than in the DSG group (21.5 [22.86] days vs. 34.7 [33.73] days, *p* = 0.0003, Wilcoxon rank-sum test). The mean number of days with unscheduled bleeding and spotting decreased over time and was lower in the DRSP group than in the DSG group in each reference period and the difference was statistically significant (Table 5).

**Table 5:** Number of days with unscheduled bleeding and/or spotting by treatment period

Cycle		DRSP 4 mg (*N* = 858)	DSG 0.075 mg (*N* = 332)	Total (*N* = 1190)	Wilcoxon rank-sum test *p* value
Cycles 2–4	*n*	527	222	749	
	Mean (SD)	9.6 (11.58)	16.9 (16.93)	11.7 (13.80)	< 0.0001
	Median	5.0	12.0	7.0	
	Min/max	0/66	0/79	0/79	
Cycles 5–7	*n*	423	157	580	
	Mean (SD)	7.4 (9.53)	10.6 (12.69)	8.3 (10.56)	0.0232
	Median	4.0	7.0	4.0	
	Min/max	0/67	0/61	0/67	
Cycles 7–9	*n*	374	137	511	
	Mean (SD)	7.2 (8.85)	10.8 (13.34)	8.2 (10.35)	0.0277
	Median	4.0	7.0	4.0	
	Min/max	0/51	0/83	0/83	
Cycles 2–6	*n*	422	172	594	
	Mean (SD)	13.7 (15.98)	23.7 (24.69)	16.6 (19.44)	< 0.0001
	Median	7.0	17.0	9.5	
	Min/max	0/89	0/134	0/134	
Cycles 2–9	*n*	305	116	421	
	Mean (SD)	21.5 (22.86)	34.7 (33.73)	25.1 (26.92)	0.0003
	Median	14.0	26.0	16.0	
	Min/max	0/95	0/156	0/156	

*N* number of patients in specified treatment group, *n* number of patients with data available, *SD* standard deviation

From cycle 2 to cycle 9, the proportion of subjects who had no bleeding or spotting increased from 30.3 to 43.7% in the DRSP and from 26.0 to 54.7% in the DSG group.

The percentage of women with frequent bleeding gradually decreased over time from 9.1% during cycles 2–4 to 5.3% during cycles 7–9 in the DRSP group and from 7.2 to 4.4% in the DSG group and was comparable between the treatment groups in each reference period. The percentage of women who experienced prolonged bleeding decreased from 12.1% during cycles 2–4 to 2.9% during cycles 7–9 in the DRSP group and from 16.7 to 10.9% in the DSG group. The incidence of prolonged bleeding in each reference period was lower in the DRSP than in the DSG group, with statistically significant differences between the groups in the second and in the third reference period (Table 6).

**Table 6:** Number of patients with prolonged bleeding per reference period

Cycle	DRSP 4 mg *n*/*m* (%)	DSG 0.075 mg *n*/*m* (%)	Difference (95% CI)	Chi square test *p* value
Cycles 2–4	64/527 (12.1)	37/222 (16.7)	− 4.52 (− 10.2; 1.12)	0.0980
Cycles 5–7	26/423 (6.1)	19/157 (12.1)	− 5.96 (− 11.5; − 0.36)	0.0172
Cycles 7–9	11/374 (2.9)	15/137 (10.9)	− 8.01 (− 13.5; − 2.51)	0.0003

*n* number of patients with data available, % percentage based on *m*, *m* number of patients in respective cycle, *CI* confidence interval

A trend toward less bleeding/spotting days was observed over time. The mean (SD) number of bleeding or spotting days decreased from 13.1 (13.05) days during cycles 2–4 to 9.7 (10.39) days during cycles 7–9 in the DRSP and from 16.9 (16.93) to 10.8 (13.34) days in the DSG group. The median number of bleeding or spotting days decreased from 10.0 to 6.0 days in the DRSP and from 12.0 to 7.0 days in the DSG group, respectively.

The number of bleeding/spotting days was lower in the DRSP than in the DSG group at all defined treatment periods. The difference between the mean (SD) number of bleeding or spotting days was statistically significant during the first reference period (cycle 2–4): 13.1 (13.05) days in the DRSP versus 16.9 (16.93) days in the DSG group (*p* = 0.0149, Wilcoxon-rank-sum-test).

### Incidence of TEAEs based on abnormal vaginal (or uterine) bleeding

In total, 46 (5.4%) of the DRSP group and 31 (9.3%) of the DSG group women experienced bleeding-related TEAEs, the majority of which were considered at least possibly related to the investigated products. Most bleeding TEAEs were of mild or moderate severity, whereas TEAEs of severe intensity were reported for four DRSP and three DSG group women.

The rate of women who withdrew from the study due to bleeding-related adverse events was 27 patients (3.3%) in the drospirenone group and 22 patients (6.6%) in the desogestrel group (*p* < 0.05).

## Discussion

Increasing satisfaction with contraception is important to help women feel comfortable with the method and continue its use. The most common reason for stopping a contraceptive method completely due to dissatisfaction is the bleeding profile. Discontinuation rates vary based on the method of birth control, with LARCs having the highest satisfaction and lowest discontinuation rate [13–15].

This study proved the superiority of drospirenone versus desogestrel even though the regimen of both contraceptives used in this trial was different: drospirenone was administered for 24 days followed by a 4-day hormone-free interval, whereas desogestrel was administered for 28 days without any interval. Therefore, subjects who received drospirenone experienced both scheduled and unscheduled bleeding, whereas the users of desogestrel experienced unscheduled bleeding only. Overall, the study results confirm the results by Archer et al. [2].

In comparison to desogestrel, the pattern with drospirenone showed less bleeding in terms of bleeding/spotting days and episodes, and the contribution of scheduled bleeding days (as opposed to spotting days) to these. Previous studies report comparable differences between ovulation inhibition and hormonal values with drospirenone versus desogestrel [16, 17]. The desogestrel group was characterized by a relatively high proportion of the bleeding pattern variables amenorrhea, infrequent bleeding, frequent bleeding and prolonged bleeding when compared to the group taking drospirenone. The percentage of women discontinuing treatment because of irregular bleeding was higher in the desogestrel group and even lower or like that of COC irrespective of whether used continuously or not [18]. The current study demonstrated that with increased treatment duration, amenorrhea and infrequent bleeding, i.e., less bleeding, became more common. This phenomenon was also observed in the desogestrel collaborative study [12].

The number of bleeding/spotting days decreased, as well as the number of bleeding/spotting episodes. At the same time, the proportion of subjects who had no bleeding or spotting increased from 30.3 to 43.7% subjects in the drospirenone and from 26.0 to 54.7% in the desogestrel group. Taken together, the bleeding became lighter and shorter in both groups, with an increasing number of subjects reporting absence of bleeding.

In addition, the clinical contraceptive efficacy of this new DRSP-only pill is similar to those COC containing DRSP and/or to the POP containing desogestrel [19].

All in all, the new DRSP-only pill will enhance compliance as the bleeding profile is improved widening the group of women able to use this contraceptive method.

## Electronic supplementary material

Below is the link to the electronic supplementary material.

Supplementary material 1 (DOCX 48 kb)
