# Correction to: A multicenter, double-blind, randomized trial on the bleeding profle of a drospirenone-only pill 4 mg over nine cycles in comparison with desogestrel 0.075 mg

**DOI:** 10.1007/s00404-020-05544-z

**Published:** 2020-04-23

**Authors:** Santiago Palacios, Enrico Colli, Pedro-Antonio Regidor

**Affiliations:** 1 Instituto Palacios, Salud Y Medicina de La Mujer, C/Antonio Acuña, 28009 Madrid, Spain; 2 Exeltis HealthCare Madrid, C/ Manuel Pombo Angulo 28, 4th Floor, 28050 Madrid, Spain; 3 Exeltis Europe, Adalperostr. 84, 85737 Ismaning, Germany

## Correction to: Archives of Gynecology and Obstetrics (2019) 300:1805–1812 https://doi.org/10.1007/s00404-019-05340-4

The article A multicenter, double-blind, randomized trial on the bleeding profle of a drospirenone-only pill 4 mg over nine cycles in comparison with desogestrel 0.075 mg, written by Santiago Palacios, Enrico Colli and Pedro-Antonio Regidor, was originally published Online First without Open Access. After publication in volume 300, issue 6, page 1805–1812 the author decided to opt for Open Choice and to make the article an Open Access publication. Therefore, the copyright of the article has been changed to © The Author(s) 2020 and the article is forthwith distributed under the terms of the Creative Commons Attribution 4.0 International License (https://creativecommons.org/licenses/by/4.0/), which permits use, duplication, adaptation, distribution and reproduction in any medium or format, as long as you give appropriate credit to the original author(s) and the source, provide a link to the Creative Commons license, and indicate if changes were made.

The original article has been revised.

