# Supplementary material for: A multicenter, double-blind, randomized trial on the bleeding profile of a drospirenone-only pill 4 mg over nine cycles in comparison with desogestrel 0.075 mg
Source: Arch Gynecol Obstet. 2019 Nov 14;300(6):1805–12. doi: 10.1007/s00404-019-05340-4 (PMC7174261; doi:10.1007/s00404-019-05340-4)
Supplement: Supplementary file 1 — Supplementary material 1 (DOCX 48 kb) [file 404_2019_5340_MOESM1_ESM.docx]

# 16.1.3. List of IECs or IRBs - Representative Written Information for Subject and Sample Consent Forms

**Austria (Central IEC)**

Site 151

Ethikkommission der Medizinischen Universität Wien Borschkegasse 8b/E06

1090 Wien

Phone: +43 (01) 40400-2147, -2248, -2241

Fax: +43 (01) 40400-1690

E-mail: [ethik-kom@meduniwien.ac.at](mailto:ethik-kom@meduniwien.ac.at)

# Czech Republic (Central IEC)

Sites 252, 254, 255, 257, 260, 262, 263, 264, 265, 266

Multicentrická etická komise Fakultní nemocnice v Motole Chair: Vratislav Šmelhaus, MD

V úvalu 84

150 06 Praha 5 - Motol

Phone: + 420 224 431 195, + 420 224 431 197,

Fax: + 420 224 431 196

E-mail: [etickakomise@fnmotol.cz](mailto:etickakomise@fnmotol.cz)

Site 251

Etická komise, Centrum neurologické péče, s.r.o. Chair: MUDr. Radomír Štrupl

Jiráskova 1389

51601 Rychnov nad Kněžnou Phone: +420 491 112 724

Fax: +420 491 112 725

E-mail: [helena@neurol.cz](mailto:helena@neurol.cz)

Site 253, 261

Etická komise Fakultní nemocnice Brno Chair: PharmDr. Šárka Kozáková Jihlavská 20

62500 Brno

Phone: +420 532 232 798

Fax: +420 547 211 961

E-mail: [etickakomise@fnbrno.cz](mailto:etickakomise@fnbrno.cz)

Site 256

Etická komise Fakultní nemocnice Ostrava Chair: MUDr. Luděk Rožnovský, CSc.

17. listopadu 1790 70852 Ostrava-Poruba

Phone: +420 597 372 542

Fax: +420 597 374 801

E-mail: [eticka.komise@fno.cz](mailto:eticka.komise@fno.cz)

Site 259

Etická komise Fakultní nemocnice Olomouc Chair: Doc. MUDr. Vladko Horčička, CSc.

1. P. Pavlova 6 77520 Olomouc

Phone: +420 588 442 477

Fax: +420 588 442 477

E-mail: [iveta.sudolska@fnol.cz](mailto:iveta.sudolska@fnol.cz)

# Germany

Site 351 (leading IEC)

Landesamt für Gesundheit und Soziales Berlin, Geschäftsstelle der Ethik-Kommission des Landes Berlin

Contact person: Dr. Christian von Dewitz

Fehrbelliner Platz 1

10707 Berlin

Phone: +49 30 90229 1220

Fax: +49 30 9028 3383

E-mail: [ethik-kommission@lageso.berlin.de](mailto:ethik-kommission@lageso.berlin.de)

Sites 353, 361, 362

Ethik-Kommission der Ärztekammer Hamburg Chair: Karin Küchenmeister

Humboldtstr. 67 a

22083 Hamburg

Phone: +49 40 20 22 99 240

Fax: +49 40 20 22 99 410

E-mail: [ethik@aekhh.de](mailto:ethik@aekhh.de)

Site 354

Ethikkommisson bei der Sächsischen Landesärztekammer Schützenhöhe 16

01099 Dresden

Phone: +49 351 8267 333

Fax: +49 351 8267 412

E-mail: [ethik@slaek.de](mailto:ethik@slaek.de)

Site 358

Ethikkommission zur Beurteilung medizinischer Forschung am Menschen Contact person: Kai Bogs

Berliner Allee 20

30175 Hannover

Phone: +49 511 380 2208

Fax: +49 511 380 2119

E-mail: [ethikkommission@aekn.de](mailto:ethikkommission@aekn.de)

Site 360

Ethik-Kommission der Bayerischen Landesärztekammer Contact person: Mrs. Schulz Kuhn

Mühlbaurstraße 16

81677 München

Phone: +49 89 4147 165

Fax: +49 89 4147 280

E-mail: [ethikkommission@blaek.de](mailto:ethikkommission@blaek.de)

Site 365

Ethikkommission des Landes Sachsen-Anhalt, Geschäftsstelle Contact person: Henning Richter

Kühnauer Straße 70

06846 Dessau -Roßlau

Phone: +49 340 65 01 291

Fax: +49 340 65 01 199

E-mail: [ek@lav.ms.sachsen-anhalt.de](mailto:ek@lav.ms.sachsen-anhalt.de)

Site 370

Ethikkommission der Landesärztekammer Hessen Contact person: Gisela Gerke

Im Vogelsgesang 3

60488 Frankfurt/Main

Phone: +49 69 97672 0,

Fax: + 49 69 97672 128,

E-mail: [info@laekh.de, info@laekh.de](mailto:info@laekh.de)

Site 371

Ethikkommission der Landesärztekammer Baden-Württemberg Contact person: Anna Gola

Jahnstr. 40

70597 Stuttgart

Phone: +49 711 769 89 0

Fax: +49 711 769 89 50

E-mail: [info@laek-bw.de](mailto:info@laek-bw.de)

# Hungary

Site 451

Metropolitan Municipality St. John's Hospital and Corporated Hospitals of North Buda, Tudományos Bizottság/Research Committee

Chair: Prof. Dr. András Jánosi Diós árok 1-3.

1125 Budapest

Site 452

Intézményi Kutatásetikai Bizottság/Institutional Research Ethics Committee Chair: Dr. Ildikó Rosta

Balassi Bálint str. 16 3000 Hatvan

Sites 453, 455, 459

Ferencvárosi Egészségügyi Szolgáltató Kiemelkedően Közhasznú Nonprofit Kft. Intézményi Kutatásetikai Bizottság/Institutional Research Ethics Committee

Chair: Dr. Imre Guller Mester u. 45.

1095 Budapest

Sites 454

Markhot Ferenc Hospital Health Provider Nonprofit Advanced Utility Kft., Intézményi Kutatásetikai Bizottság/Institutional Research Ethics Committee

Chair: László Hernádi Széchenyi u. 27-29.

3300 Eger

Site 457

Komárom -Esztergom County St. Borbala Hospital, Intézményi Kutatásetikai Bizottság/Institutional Research Ethics Committee

Chair: Dr. Gábor Nagy Dózsa György str. 77. 2800 Tatabánya

Site 460

Institutional Ethics Commitee of II. Rákóczi Ferenc Hospital Chair:

Kassai út 45-49

3800 Szikszó

Site 461

Institutional Ethics Commitee of St. John Hospital and North-Buda United Hospitals, Chair: Prof. Dr. András Jánosi

Diós árok 1-3.

1125 Budapest

Sites 462, 463

Institutional Ethics Commitee of Ferencvárosi Health Provider Nonprofit Advanced Public Utility Kft.

Chair: Dr. Imre Guller Mester u. 45.

1095 Budapest

Site 464

Institutional Ethics Commitee of Józsefvárosi Health Provider Kft. Auróra u. 22-28

1084 Budapest

Lead EC

Medical Research Council

Ethics Committee for Clinical Pharmacology Chair: Prof. Dr. Zsuzsanna Fürst

Phone contact: Istvánné Magyari Arany János u. 6-8.

1051 Budapest

Phone: +36 1 7951195

Fax: +36 1 7950-168

E-mail: [istvanne.magyari@emmi.gov.hu](mailto:istvanne.magyari@emmi.gov.hu)

# Poland (Central IEC)

Ethics Committee at Local Medical Chamber Chair: Mariusz Janikowski MD

ul. Krupnicza 11A 31-123 Kraków

Phone: +48 12 619 17 12

Fax: +48 12 422 57 55

E-mail: [akrawczyk@hipokrates.org](mailto:akrawczyk@hipokrates.org)

# Romania (Central IEC)

Comisia Nationala de Etica pentru Studiul Clinic al Medicamentului National Etichs Committee for the Drug Clinical Study

Chair: Prof. Dr. Sava Dumitrescu 011478 Bucharest

str. Aviator Sanatescu, nr.48, sector 1 Phone: +403 1405 1076

Fax: +403 1405 1075

# Spain

Lead EC, Sites 751, 755

CEIC Área 2 - Hospital Universitario de La Princesa Contact persons: Cecília López / Julio González

C/ Diego de León, 62

28006 Madrid

Phone: +34 91 520 2476

Fax: +34 91 520 2560

E-mail: [ceic.hlpr@salud.madrid.org](mailto:ceic.hlpr@salud.madrid.org)

Sites 752, 753, 762

IDIAP Jordi Gol i Gurina

Contact person: Mari Pau Moreno

Av. Gran Via de les Corts Catalanes, 591 Àtic 08007 Barcelona

Phone: +34 93 482 4572

Fax: ++34 93 482 4174

E-mail: [pmoreno@idiapjgol.org](mailto:pmoreno@idiapjgol.org)

Site 759

CEIC Hospital General de Vic - Fundació FORES Contact person: Lidia Soler

C/ Francesc Pla "El Vigatà", 1 08500 Vic

Phone: +34 93 702 7713

Fax: +34 93 885 0308

E-mail: [lsolerdelcoll@chv.cat](mailto:lsolerdelcoll@chv.cat)

Site 760

CEIC Hospital Mútua Terrassa Contact person: Susana Redondo Plaza Dr. Robert, 5

08221 Terrassa

Phone: +34 93 736 5050/Exit. 1032

Fax: +34 93736 5059

E-mail: [ceichmt@mutuaterrassa.es](mailto:ceichmt@mutuaterrassa.es)

Site 761

CEIC Capio Hospital General de Catalunya Contact person: Montse Granados

C/ Pere i Pons, 1

08195 Sant Cugat del Vallès Phone: +34 93565 6000/Exit.5077

Fax: +34 93589 2498

E-mail: [mgranado@hgc.es](mailto:mgranado@hgc.es)

# Slovakia

Lead EC, Sites 851, 857, 859 Etická komisia JLF UK

Chair: Prof. MUDr. Gabriela Nosáľová, DrSc. Sklabinská 26

03753 Martin

Phone: +421 434 132 535

Fax: +421 434 134 807

E-mail: [nasalova@jfmed.uniba.sk](mailto:nasalova@jfmed.uniba.sk)

Site 852

Etická komisia GPN

Chair: MUDr. Viera Lesná. Partizánska 27

81103 Bratislava

Phone: +421 254 640 091

Fax: +421 254 640 092

E-mail: [lesna@gpn.sk](mailto:lesna@gpn.sk)

Sites 854, 860

Etická komisia Banskobystrického samosprávneho kraja Chair: Mgr. Katarína Čupková

Námestie SNP 23

97401 Banska Bystrica

Phone: +421 484 325 570

Fax: +421 484 325 515

E-mail: [zdenka.kadasiova@vucbb.sk](mailto:zdenka.kadasiova@vucbb.sk)

Site 855

Etická komisia Bratislavského samosprávneho kraja Chair: MUDr. Valerián Potičný

Sabinovská 16

82005 Bratislava 25

Phone: +421 248 264 823

Fax: +421 248 264 386

E-mail: [katarina.molnarova@region-bsk.sk](mailto:katarina.molnarova@region-bsk.sk)

Sites 858

Etická komisia Prešovského samosprávneho kraja Chair: MUDr. Július Zbyňovský, MPH

Námestie mieru 2

08001 Prešov

Phone: +421 517 081 635

Fax: +421 517 481 638

E-mail: [lubica.cuperova@vucpo.sk](mailto:lubica.cuperova@vucpo.sk)

Subject Information Sheet and Informed Consent Form:

Final English Version 1.0, 14-DEC-2011

# SUBJECT INFORMATION SHEET

**Title: A Pivotal, Multicentre, Double-Blind, Double-Dummy, Randomised Trial on the Contraceptive Efficacy, Tolerability and Safety of LF111 (Drospirenone) Over 9 Cycles in Comparison with Desogestrel 0.075 mg**

**Protocol No. CF111/302**

**Sponsor:** Laboratorios León Farma S.A. La Vallina s/n

Polígono Industrial de Navatejera 24008 Navatejera (León), Spain

**Investigator:** Name Address

City, State Zip Phone Number(s)

Subject Number | | | | | | |

# Introduction

You are being invited to take part in a clinical research study. Before you decide to take or not to take part in this study, it is important for you to understand why the research is being done and what it will involve. This information sheet and consent form provides you with essential information about this trial and your rights as a subject in clinical research, so that you can make an informed decision about your participation.

Please take time to read the following information carefully and discuss it with friends, relatives and your general practitioner, if you wish. Please ask us if there is anything that is not clear or if you would like to receive more information. Take time to decide whether or not you wish to take part in this study.

# Clinical Trials

Clinical studies are necessary in order to obtain or increase knowledge on whether drugs which have not yet been registered are effective and safe. According to the law, clinical studies must be carried out before a new drug is registered. The development of new drugs and clinical studies are subject to legal regulations, recognised international guidelines (e.g. ICH-GCP guidelines) and ethical principles (Declaration of Helsinki).

Laboratorios León Farma S.A.

CF111/302 Master ICF, Final English Version 1.0, 14-Dec-2011 Page 1 of 14

# Purpose of the study

Oral contraceptives are among the most popular and most effective forms of contraception. They can be divided into combined-oral-contraceptive-pills (estrogen plus progestogen) and progestogen-only-pills. Progestogen-only-pills are especially useful for women who are intolerant to estrogens (e.g. due to migraine, high blood pressure, high cholesterol levels, obesity, diabetes or smoking habits) and for breastfeeding women.

Drospirenone (DRSP) is a synthetic progestogen with a pharmacological profile close to natural progesterone. In combination with ethinyl estradiol DRSP is part of many oral contraceptives (e.g. Yasmin^®^, Yasminelle^®^, YAZ^®^) that are widely used in Europe and in the USA. In addition, DRSP in combination with estradiol (Angeliq^®^) is used in Europe and in the USA as hormone replacement therapy for managing menopausal symptoms.

So far experience with DRSP given alone is limited. In a small trial DRSP alone was shown to suppress ovulation at 4 mg per tablet/day. The new drug LF111 used in this trial contains 4 mg DRSP per tablet/day. It is expected that LF111 is a reliable method of contraception.

The aim of this clinical research trial is to show the efficacy and safety of LF111 as an oral contraceptive and to compare its tolerability to that of desogestrel 0.075 mg (trade name: Cerazette^®^).

The trial will be conducted in 8 European countries in approximately 88 investigational sites. It is planned to include 1200 women in the trial for a duration of 9 medication cycles. The trial will be performed by the pharmaceutical company (sponsor) Laboratorios León Farma S.A in Spain.

# Participation is voluntary

It is entirely up to you whether you take part or not. You may withdraw from the trial at any time, for any reason without penalty or loss of benefits to which you are otherwise entitled. If you do not wish to take part in this trial or later withdraw from it, this will not affect your future care or your relationship with your doctor in any way.

New information may become available during the course of the trial that might affect your willingness to continue in the study. If this happens, your trial doctor will explain it to you as soon as possible and discuss with you whether you want to continue participation in the study. If you decide to withdraw, your trial doctor will make arrangements for your future care. In addition, it may happen during the trial that for your own safety your trial doctor withdraws you from the study. If this happens, he/she will explain the reason and arrange for your care to continue. In case of withdrawal from the study, you may request that no further data will be collected and added to the trial database. If you want this to be done, please contact your trial doctor. In the event of withdrawal of consent to the study, the data and laboratory samples already collected up to this time point cannot be deleted with respect to the legal requirement to store the data.

You may also be withdrawn from the trial if you do not follow the trial doctor’s directions or if your health condition changes in a way that staying in the trial may risk your health or the outcome of the trial. The entire trial can also be discontinued at any time by the trial doctor, Laboratorios León Farma S.A. or by any regulatory authority if the safety of subjects is found to be at significant risk.

# Trial treatment

In this trial the subjects will receive either LF111 or desogestrel 0.075 mg.

LF111 tablets (white) contain 4 mg drospirenone (DRSP), given for 24 days followed by a 4-day interval of green tablets without any active ingredient. LF111 is a new medication which will be investigated during the study (the test drug).

Desogestrel 0.075 mg is a progestogen-only-pill that is available on the market, i. e. with already established efficacy and safety (the reference drug). Each of the 28 tablets contain active ingredient.

# Trial procedures

Including the screening visit (V1a) at the beginning and the final visit at the end (V6), your trial participation will last approximately 39 weeks in total. A randomisation visit (V1b) and five control visits will be performed (visits V2 to V4 between day 22 and 26 of medication cycles 1, 3, and 6; visit V5 between day 29 and 31 of medication cycle 9; and visit V6 seven to ten days after the last trial medication intake).

During visit V1a your doctor will discuss with you the participation in the trial and will ask for your informed consent. He/she will check whether there is any medical or other reason that you may not participate. Therefore it is very important that you answer to all questions regarding your medical history, your gynaecological history and the medications you are taking. Questions will be for example regarding your last bleeding, the regularity of your bleeding, whether you use any other contraceptive method and if you suffer from any cycle-related complaints.

Your doctor will measure your pulse rate, blood pressure, height and weight, and he/she will perform a general physical and a gynaecological examination. You will be asked for a urine sample for a pregnancy test and test of urine parameters. A blood sample of approximately 20 ml, which is equal to four teaspoons, will be taken for analysis.

You have to stop any other currently used contraceptive method and not to start further contraceptives during the study. There are some exceptions to this rule explained in the following sections, when the use of an additional method like condoms or diaphragm is recommended.

When the results of the laboratory tests are available and if all conditions for participation are fulfilled you will come to visit V1b. You will be randomly assigned (by chance, with a computer procedure similar to throwing dice) to one of two treatment groups:

1. LF111

2. Desogestrel 0.075 mg

You have a 71% (5 out of 7) chance to receive LF111 and a 29% (2 out of 7) chance to receive desogestrel 0.075 mg.

This treatment will be double-blind and double-dummy. That means that neither you nor your doctor will know to which group you will be allocated. You will be given two blisters for consecutive intake once daily. One of them will look like LF111 and the other like desogestrel 0.075 mg. However, only one of them will contain the active substance. The other blister will be filled with placebo pills only (i. e. without any active ingredient).

In any case you will receive an active contraceptive - either from the test or from the reference blister.

# Each day you will have to take one pill from blister A and one pill from blister B.

You will have to take the pills at the same time each day.

Your site trial team will give to you the first packages with trial medication (for 2 cycles), an electronic diary (e-diary) and a subject card. In the e-diary you are asked to enter daily all information regarding the intake of the trial medication, the occurrence of vaginal bleedings and the use of additional contraceptive methods, as well as your sexual activity at the end of each cycle. Your doctor will explain how to use the e-diary. **Please bring the e-diary to each visit.**

You will also be provided with home pregnancy test kits and will be asked to perform a pregnancy test at home at the beginning of each new medication cycle.

For Germany and Austria only: If you have been chosen for the special blood tests, a blood sample of approximately 12 ml, which is equal to two teaspoons, will be taken for analysis. **You have to come to visits V1b and V5 in the morning in fasting condition.**

For Germany and Czech Republic only: An electrocardiogram (ECG) will be performed. This is a painless examination that looks at the electrical activity of your heart, and it

takes about 5 minutes.

If you continue in the study, at visits V2 to V4 your doctor will hand over the following packages with trial medication and home pregnancy test kits to you. **During the trial you should bring the trial medication with you at each visit for inspection – also empty packages and blisters.**

At all following visits (except V6) your doctor will check the entries you have made in your e-diary and may ask you for more explanations. Please carry the subject card with you at all times. During each visit the next appointment with your trial doctor will be noted on the card. Your subject card will be collected at visit V6.

Diagram of the course of the trial with all examinations:

| **Visits** | **V1a (Screening)** | **V1b (Randomisation)** | **V2** | **V3** | **V4** | **V5^1^** | **V6**  **(Follow-Up)** |
| --- | --- | --- | --- | --- | --- | --- | --- |
| **Medication cycle** |  |  | **1** | **3** | **6** | **9** | **7-10 days after last pill intake** |
|  |  |  | **Day 24 ± 2**  **of medication cycle** | | | **Day**  **29 + 2** |  |
| Physical/gynaecological  examination^2^ | x |  |  |  |  | x |  |
| Blood pressure, pulse, body weight;  height (V1a only) | x |  | x | x | x | x |  |
| Electrocardiogram^3^ |  | x |  |  |  | x |  |
| Blood sample for safety  laboratory tests^4^ | x |  |  | x | x | x |  |
| Blood sample for  special laboratory tests, in fasting condition^3,4^ |  | x |  |  |  | x |  |
| Urine sample^5^ | x |  |  | x |  | x |  |
| Pregnancy test (blood) | x |  |  |  |  | x |  |
| Pregnancy test (urine) |  |  | x | x | x |  | x |
| E-diary hand out |  | x |  |  |  |  |  |
| Medication and home  pregnancy test hand out |  | x | x | x | x |  |  |
| Bring e-diary to the site |  |  | x | x | x | x |  |
| Bring medication to the  site (incl. empty blisters) |  |  | x | x | x | x |  |

1. or Early Discontinuation Visit
2. Gynaecological examination: inspection of the external genital organs, a speculum examination, a cervical swab, the palpation of the internal genital organs and the examination of your breasts.

Additionally, an intravaginal ultrasound will be performed.

1. Not in all subjects
2. Blood tests for: Thyroid-stimulating hormone (TSH), haemoglobin, red blood cell count, mean corpuscular volume (M.C.V.) and associated parameters, haematocrit, M.C.H., white blood cell count, differential white blood cell count including neutrophils, lymphocytes, eosinophils, basophils and monocytes, platelet count. sodium, potassium, chloride, creatinine, blood urea nitrogen (BUN), calcium, glucose, total proteins, albumin, total cholesterol (HDL, LDL cholesterol), triglycerides, gamma glutamyl transferase, total and direct bilirubin, alkaline phosphatase (ALP), alanine aminotransferase (ALAT), aspartate aminotransferase (ASAT), creatine phosphokinase (CPK), lactate dehydrogenase (LDH).

Special blood test (not in all subjects): Haemostatic variables: factor VIIc, factor VIIIc, protein C activity, antithrombin III activity, D-dimer. Carbohydrate metabolism: fasting plasma glucose, serum insulin, C-peptide. Bone metabolism: bone alkaline phosphatase, cross-linked c-terminal telopeptides (CTX)

1. Urine tests for: Protein, glucose, ketones, blood, pH, leukocytes, nitrite, urobilinogen, bilirubin, haemoglobin – dipstick. If any of the measured parameters of urine analysis is out of range/pathologic, sample will be shipped to the laboratory for microscopic examination.

# How to take the trial medication

If you start intake of an oral contraceptive newly, you have to start the intake of the trial medication on the first day of your next menstrual bleeding. The doctor will explain it to you in detail.

If you switch directly from another contraceptive pill you have to take the first tablets of the trial medication on the day following the last active tablet of your previous hormonal contraceptive.

You will receive cardboard wallets with trial medication for each cycle. A wallet contains two blisters (A and B). **Each day you will have to take one pill from blister A and one pill from blister B.**

Blister A contains 24 white tablets and 4 green tablets, which are numbered 1-28 and must be taken in this order. On the blister card there are arrows starting in the left upper corner leading you through the cycle. **To assure reliable contraception throughout the medication cycles it is very important to follow the sequence indicated by the arrows and to take the green tablets at the end of the cycle as indicated and not at the beginning or in between.**

Blister B contains 28 white tablets. Please follow the direction of the arrows on the blister.

Please enter date and week day of first intake in the fields provided.

The tablets should be swallowed whole at the same time every day. There is no break between two medication cycles, i. e. you start with the next medication wallet directly after finishing the previous medication wallet. The intake of trial medication in this manner will continue for 9 medication cycles. Your vaginal bleeding pattern may change under trial medication. Nevertheless you should continue the intake of trial medication as planned.

# Please make sure that you are familiar with the intake schedule after having received the tablets and feel free to ask your doctor if anything remains unclear.

If you have forgotten to take the trial medication at the usual time, it must be taken within the **following 12 hours** at the latest and the next tablets should be taken at the usual time.

If you are more than 12 hours late in taking your trial medication, effective contraception may be reduced for the next 7 days. In such a case, take it as soon as you remember - at the latest together with the next usual pills. Take the next tablets on time, and continue taking the remaining tablets from the current blister as scheduled. **Additional contraceptive methods (e.g. a condom or a diaphragm) should be used for the next 7 days.**

In case more tablets have been forgotten your doctor will advise you how to correctly re- enter the medication cycle so that each medication cycle has a length of 28 days.

# What must I take into consideration?

When you gave birth to a child recently you can only participate if you are not breastfeeding.

The contraceptive effectiveness of the trial medication may be disturbed by the concomitant administration of other medications that increase the degradation of steroid hormones so that the hormones are not as effective as usual. These medications are for example liver-enzyme inducing drugs, such as barbiturates, rifampicin, griseofulvin, phenylbutazone and antiepileptic agents. Reduced efficacy of the trial medication can also be expected when using broad spectrum antibiotics such as ampicillin or tetracyclines concomitantly (due to changes of the intestinal flora by the antibiotics), St. John’s worth or after the ingestion of activated charcoal (3 hours before or after intake of the charcoal).

The trial medication can also increase potassium blood level. Other drugs may also increase potassium blood level like certain pain medications (NSAIDs, such as ibuprofen, naproxen), spironolactone, potassium supplementation, ACE inhibitors, angiotensin-II receptor antagonists and heparin.

Therefore, if you take any concomitant medication apart from the trial medication, please tell your doctor so that he/she can advise you if substances of the above mentioned groups are contained and whether further contraceptives measures like condoms or diaphragm have to be used during this medication cycle.

Insulin or oral antidiabetic requirements may be altered due to an influence of oral contraceptives on glucose tolerance.

In the event of vomiting or intestinal disease like diarrhoea the tablet intake should not be interrupted. Additional contraceptive methods (e.g. a condom or a diaphragm) should be used for the next 7 days if vomiting or diarrhoea has taken place in the first 3 to 4 hours after intake of the trial medication.

# Please note that oral hormonal contraceptives do not protect you from infection with HIV or other sexually transmitted diseases.

If you become pregnant or suspect that you are pregnant during this clinical trial please inform your doctor immediately. If the pregnancy is confirmed, you have to stop the trial medication immediately and your doctor will follow-up pregnancy, birth and the condition of your child. Also pregnancies within 3 months after (regular or premature) termination of the trial have to be reported to the trial doctor. If you stop your participation earlier because you want to get pregnant you are kindly asked to inform your doctor as soon as you become pregnant within one year following the study.

# Risks and Discomforts

*Trial drug*

LF111 is a new contraceptive formulation and there is a certain risk that it might be not as effective as other oral contraceptives that are already on the market (such as desogestrel 0.075 mg).

As any other drugs, both trial medications have the potential to cause side effects. Their influence on your health can vary from symptoms that cause you mild discomfort to more severe conditions that will require treatment.

The side effect profile of DRSP (LF111) given alone is not fully elucidated. When given in combination with ethinyl estradiol or estradiol the most common events that have been reported in more than 1% of subjects in the pivotal clinical studies include, which may or may not be drug-related:

- - Upper respiratory infection, headache, breast pain, vaginal yeast infection, vaginal discharge (leukorrhea), diarrhea, nausea, vomiting, vaginitis, abdominal pain, flu syndrome, painful menstruation (dysmenorrhea), allergic reaction, urinary tract infection, accidental injury, bladder infection (cystitis), tooth disorder, sore throat (pharyngitis), infection, fever, surgery, sinusitis, back pain, emotional lability, migraine, Pap smear (microscopic examination of cervical cells) suspicious, indigestion (dyspepsia), rhinitis, acne, gastric flu (gastroenteritis), bronchitis, skin disorder, intermenstrual bleeding, libido decreased, weight gain, pain, depression, cough increased, dizziness, menstrual disorder, pain in extremity, pelvic pain, and weakness (asthenia).
  - The use of combination oral contraceptives is associated with increased risks of several serious conditions including venous and arterial thrombotic and thromboembolic events (such as myocardial infarction, thromboembolism, stroke), liver tumor, gallbladder disease, and high blood pressure. The risk of serious morbidity or mortality is very small in healthy women without underlying risk factors. The risk of morbidity and mortality increases significantly in the presence of other underlying risk factors such as high blood pressure, high cholesterol levels, obesity and diabetes.

The following side effects have been reported for desogestrel 0.075 mg:

- - Common (affecting less than 1 in 10 women): Mood changes, decreased sexual drive (libido), headache, nausea, acne, breast pain, irregular or no periods, weight increase.
  - Uncommon (affecting less than 1 in 100 women): Infection of the vagina, difficulties in wearing contact lenses, vomiting, hair loss, painful periods, ovarian cysts, tiredness.
  - Rare (affecting less than 1 in 1000 women): Skin conditions such as: rash, hives, painful blue-red skin, lumps (erythema nodosum)

If you do not understand the meaning of any of these side effects or if you wish to have a more detailed description of these conditions and the risks associated with each one, please ask your trial doctor.

If any side effect occurs, it can either be treated by your doctor or will disappear after discontinuation of the trial medication. In case of unusual or severe symptoms please consult your trial doctor immediately. The symptoms will be noted and closely monitored by the trial doctor. Also he/she will decide whether it is necessary to stop taking the trial medication.

Breast cancer

It is important to regularly check your breasts and you should contact your doctor if you feel any lump in your breasts. Breast cancer has been found slightly more often in women who take birth-control pills than in women of the same age who do not take them.

Breast cancer is rare under 40 years of age but the risk increases as the woman gets older. Therefore, the extra number of breast cancers diagnosed is higher if a woman takes the pill when she is older. How long she takes the pill is less important.

- - In 10 000 women who take the pill for up to 5 years but stop taking it by the age of 40, there would be 20 extra cases in addition to the 160 cases normally diagnosed.

It is not certain whether birth-control pills cause an increased risk of breast cancer. It may be that the women were examined more often, so that the breast cancer is noticed earlier.

Thrombosis

Thrombosis is the formation of a blood clot, which may block a blood vessel. A thrombosis sometimes occurs in the deep veins of the legs (deep venous thrombosis). If this clot breaks away from the veins where it is formed, it may reach and block the arteries of the lungs, causing a so-called “pulmonary embolism”. People do not always fully recover from a thrombosis, and very rarely, they are fatal.

Deep venous thrombosis is a rare occurrence. It can develop whether or not you are taking birth-control pills. It can also happen if you become pregnant. The risk is higher in pill-users than in non-users. The risk with progestogen-only pills like the trial medication is believed to be lower than in users of pills that also contain estrogens (combined-oral- contraceptive-pills).

Contact your doctor as soon as possible if

- - you notice possible signs of a blood clot (e.g. severe pain or swelling in either of your legs, unexplained pains in the chest, breathlessness, an unusual cough, especially if you cough up blood);
  - you are to be immobilised or are to have surgery (consult your doctor at least four weeks in advance);

During the intake of the trial medication occasional irregular bleeding or absence of bleeding may occur, and there could be other side effects not described in this subject information sheet. Irregular bleeding occurs most often during the first few months of oral contraceptive use. The irregular bleedings are usually temporary and of no medical significance. If they occur, please continue taking the tablets regularly. If you consider your bleeding to be abnormal (e.g. more intense or longer than your regular menstrual bleeding), you should, however, consult your doctor.

As with any drug at any stage of development, there is always a risk of rare or previously unknown side effects developing from treatment. Your trial doctor will inform you of any significant new findings that may occur during the course of this research, which may affect your decision to remain in the study.

Please show your subject card, which documents that you are participating in this study, at all times in case a medical treatment is necessary at some other location (e.g. accident or emergency). If treated by another doctor, please inform your trial doctor as soon as possible.

*Pregnancy and birth defects*

If you do become pregnant during the study, there may be risks to the unborn child and yourself, which are currently unknown. If you suspect that you have become pregnant, you must notify the trial doctor immediately.

*Blood withdrawals*

When a blood sample is taken, there may be minimal discomfort and bruising at the site where the needle is inserted through the skin. There is a possibility of fainting and bruising or infection at the insertion point.

*Physical Examinations*

The intravaginal ultrasound examination may cause a little discomfort for you, as may the speculum examination, the palpation of your internal organs and the examination of your breast, but they usually do not hurt.

# Potential benefits

The potential benefit of being in this trial is that you will get a pill for free for up to 9 cycles (8 months). Besides, your health will be monitored very closely and carefully during the study, e.g. you will have profound gynaecological examinations from a qualified doctor during the study. Additionally you might benefit from taking a progestogen-only-pill especially when you are intolerant to estrogens that are part of combined pills.

# Costs during trial participation

You do not have to pay for the participation in the trial.

# Alternative treatment

The following alternative contraceptive treatments are available: other birth control pills, intrauterine devices with or without an additional hormone, condoms, implants, injectable hormones, diaphragms, spermicides, female condoms, cervical caps, contraceptive sponges etc. Also so called natural methods, e.g. the temperature method, are known. All mentioned methods differ in their reliability to prevent pregnancies, their risks and their advantages. Please ask your trial doctor if you need more information.

# Ethics

This trial is subject to international guidelines and regulations: the Declaration of Helsinki (1996), the rules for Good Clinical Practice (GCP) and the national laws. Your rights as a volunteer in a clinical trial are laid down in these regulations. The protocol for the trial has been reviewed and approved by an independent ethics committee.

# Insurance

Laboratorios León Farma S.A. has taken out insurance coverage in accordance with the requirements in your country.

[name and address of insurance company and policy no., if required]

Any impairment of health which may be due to participation in this clinical trial must be reported to the insurer without delay. Thus, you must report any such impairment without delay to your doctor conducting the study. In terms of the insurance policy, and as a participant in this study, please discuss any medical treatment for any of these impairments with your doctor prior to receiving treatment. The only exceptions are emergency cases requiring immediate treatment, in which case the doctor conducting the trial should be notified immediately. Unwanted pregnancy does not represent damage to health in the context of the subject insurance. Thus, there will be no compensation for any unwanted pregnancy and for malfunctions or malformations of the unborn/new born baby.

A copy of the general insurance conditions for a possible injury/disablement that occurs while taking part in this trial can be obtained from your trial doctor.

# Your obligation as a trial participant is to:

1. Provide a complete medical history and answer all questions truthfully.
2. If you plan to undergo any other medical treatment during this clinical study, or are taking any medications, you must disclose this to the trial doctor in advance.
3. Attend all visits and complete the electronic diary daily.
4. If you suffer from any injury or unexpected reaction to the medication you receive as a part of this research study or if you think you are pregnant you must notify the trial doctor immediately and seek treatment in accordance with the direction of the trial doctor.
5. To take trial medication as required, returning medication and packaging (including empty blister cards) at each trial visit.
6. To perform a pregnancy test at home at the beginning of each medication cycle. Further, you may not participate in another clinical trial at the same time.

By signing this consent form you are not giving up any of your legal rights as a trial participant.

# Confidentiality

By taking part in this study, you consent that only the information that is necessary for the analysis and evaluation of the trial will be collected by Laboratorios León Farma S.A. and its authorised representatives. The information will not identify you by name but by your subject number (6 digits) and your year of birth. If the results of the trial are published, your identity will remain confidential.

At the end of the study, your trial data will be kept by Laboratorios León Farma S.A.. Current guidelines require that all trial documentation related to the conduct of a clinical trial must be retained for the longer of (I) 2 years after the last marketing authorisation for the trial drug has been approved or the sponsor has discontinued its research with respect to such trial drug, or (II) such longer period as required by applicable regulatory requirements or (III) 15 years. Your identity information will be maintained by the trial

doctor for 15 years unless a longer period is required by applicable law or regulation. Your information will be transferred into a computer database and processed to allow the results of this trial to be analysed and reported or published. Under your national data protection law "[*Identification of national law*]" you have the right to access, through your trial doctor, to all the information collected about you and, if applicable, ask for corrections.

The laboratory samples and the cervical cytology samples will be stored until the final trial report will be issued. If necessary to better characterise the effect of the treatment, some additional analyses can be done on these samples such as a determination of hormone blood levels or coagulation factors. However, no genetic analysis will be performed on your samples.

Only authorised representatives of Laboratorios León Farma S.A., and its designees, the ethics committee that approved this trial and regulatory authorities in various countries will have direct access to your medical records. This is necessary to check that the trial is being performed correctly and that the information collected about you is accurate. All personnel accessing your records are required to respect your confidentiality at all times.

# Authorising the use of medical information

This clinical trial may only be performed by collecting and using your medical information. National and international data protection regulations give you the right to control the use of your medical information. Therefore, by signing this form you specifically authorise your medical information to be checked, transferred and processed as follows:

- - The authorised representatives of Laboratorios León Farma S.A., and its designees, the ethics committee and regulatory authorities’ inspectors in various countries may review your medical information by direct access to your medical records.
  - Trial data, including your medical information, and information on any medical samples taken, may be processed, which means it will be collected, entered into computer databases, verified, analysed, printed and reported as necessary for legitimate scientific purposes, including use in future medical or pharmaceutical research as outlined in the trial protocol.
  - Trial data may be transferred to other countries for processing, including countries not covered by the Data Protection Legislation.

# Contacts

If you have questions about the study, you have questions concerning the nature of the research or your rights as a research subject or you believe that you have sustained a research-related injury, you should contact your trial doctor:

Name:.....................................................................................................................................

Telephone:..............................................................................................................................

Subject Number | | | | | | |

**INFORMED CONSENT FORM**

**Trial Title:** A Pivotal, Multicentre, Double-Blind, Double-Dummy, Randomised Trial on the Contraceptive Efficacy, Tolerability and Safety of LF111 (Drospirenone) Over 9 Cycles in Comparison with Desogestrel 0.075 mg

**Trial Code:** CF111/302

Name of subject:

, (full name of the participant, *can be entered by the site or subject*)

hereby declares by signing this informed consent form that:

I have read the subject information sheet for this study. I have received an explanation of the nature, purpose, duration, and foreseeable effects and risks of the trial and what I will be expected to do. My questions have been answered satisfactorily. I have been given ample time to and opportunity to enquire about details of the study.

I agree to take part in this study. I agree to cooperate fully with the doctor conducting the trial and will contact him***/***her immediately if I suffer any unexpected or unusual symptoms or if I think that I am pregnant during the study. For the duration of the study, I will notify the investigator of any other medical treatments that may be necessary for me to undergo. I agree that, before final inclusion in the study, laboratory tests will be performed. If any of these results show that I am not eligible for the study, I will not be included in the study.

I have informed the doctor conducting the trial of all my previous or present illnesses and medication and of any consultation that I have had with any doctor in the last months.

I have further informed the doctor conducting the trial of any participation by me in other clinical studies in the past months.

I am aware that if I do not cooperate fully with the doctor’s requests and directions, I may harm myself by participating in the study.

I understand that I do not have to sign this consent form. I also understand if I do not sign this form, I will not be able to participate in this study.

I understand that my participation in the trial is voluntary. I understand that I may withdraw from the study:

- - Whenever I wish
  - Without having to explain why
  - Without this affecting my medical care

I further understand that any information that becomes available during the course of the trial that may affect my willingness to take part will be disclosed to me as soon as practicable.

Representatives of the sponsor, independent ethics committee, or local or foreign regulatory authorities may wish to examine my medical records to verify the information collected. By signing this document, I give permission for this review of my records.

Subject Number | | | | | | |

I understand the description in this document of the extent that my protected health information will be used or disclosed for research and for treatment in connection with research. I also understand the description in this document of the extent to which my protected health information will not be used or disclosed. I consent to the use and disclosure of my protected health information to carry out treatment for research as described in this document. I also authorise the transfer to other countries and other uses and disclosures of protected health information described in this document.

I agree to keep my code (PIN) for the electronic diary strictly confidential and not to disclose it to other persons.

**I agree that my general practitioner may be informed by the trial doctor about my participation in this study.**

YES NO

I agree that my general practitioner may send my previous medical records to the trial doctor.

YES NO

# I hereby freely consent to take part in the study.

Signature of the participant Date

Printed name of participant (*can to be entered by the subject)*

Signature of the person

administering the consent

Date

Printed name of the person administering the consent

# A copy of the signed subject information sheet and consent form must be provided to the subject.
